# Translation, adaptation, and validation of a Chinese version of the Hypertension Self-Care Activity Level effects (H-SCALE) for patients with hypertension

**DOI:** 10.1186/s12912-024-01993-y

**Published:** 2024-05-17

**Authors:** Ting-Yu Chen, Chi-Wen Kao, Shu-Meng Cheng, Chieh-Yu Liu

**Affiliations:** 1 Department of Nursing, Chang Gung University of Science and Technology, Chiayi, Taiwan; 2 School of Nursing, National Defense Medical Center, No.161, Sec. 6, Minquan E. Rd Neihu Dist, Taipei, 11490 Taiwan; 3 Division of Cardiology, Department of Internal Medicine, Tri-Service General Hospital, National Defense Medical Center, Taipei, Taiwan; 4 Department of Health Care Management, National Taipei University of Nursing and Health Sciences, Taipei, Taiwan

**Keywords:** H-SCALE, Hypertension, Lifestyle, Translation, Validation

## Abstract

**Background:**

Lifestyle modification is an essential component of prevention and management of hypertension. Existing instruments in Taiwan focus on assessing lifestyle modifications by evaluating medication adherence or confidence in controlling blood pressure. However, other self-care activities, such as diet, physical activity, weight management, smoking, and alcohol consumption are also important. The Hypertension Self-Care Activity Level Effects (H-SCALE) is one such instrument, but there are no similar tools available in Taiwan.

**Aim:**

This study aimed to translate the H-SCALE into Chinese and test its validity, and reliability in a sample of adults with hypertension.

**Methods:**

The English version of the 31-item H-SCALE was translated into Chinese using the forward-backward method. The content validity index (CVI) of the translated scale was determined by five experts in hypertension. Item analysis was conducted with a pilot sample of 20 patients with hypertension. Cronbach’s α was used to establish the internal consistency reliability for the Chinese version of the H-SCALE (H-SCALE-C). Exploratory factor analysis (EFA) explored the structure of the H-SCALE-C. Additionally, construct validity was examined with confirmatory factor analysis (CFA). Patients with hypertension were recruited by convenience sampling from a cardiovascular outpatient clinic of a medical center in northern Taiwan. A total of 318 patients met the inclusion criteria and participated in factor analysis in the study.

**Results:**

Pilot testing of the scale items indicated most patients could not accurately estimate the number of days of alcohol consumption for the previous week. Therefore, three alcohol-related items were removed. The adaptation resulted in a 28-item H-SCALE-C. EFA revealed a 4-factor solution with 13 items that explained 63.93% of the total variance. CFA indicated a good fit for a 4-factor model and construct validity was acceptable. Internal consistency reliability was acceptable (Cronbach’s alpha for the four subscales ranged from 0.65 to 0.94). Convergent validity was acceptable, and discriminant validity was significant.

**Conclusions:**

The H-SCALE-C is a valid, reliable tool for promptly assessing life-style activities for patients with hypertension in Taiwan. The instrument is suitable for assisting healthcare providers in evaluating self-care activities, which could be used to facilitate lifestyle modifications for patients with hypertension.

## Background

The World Health Organization (WHO) reported 1.28 million adults were affected by hypertension in 2021, which contributed to premature death and disability worldwide [1]. In Taiwan, the incidence of hypertension in 2019 was reported to be 25% [2]. The incidence of hypertension is expected to increase to 65.4% in 2025, with Asian populations contributing significantly to this rise due to their large numbers [1]. Lifestyle modification is essential for the prevention and management of hypertension, as it not only helps reduce the number and dosage of antihypertensive medications but also lowers the risk of cardiovascular complications [2]. Multidimensional comprehensive assessment instruments are often used to evaluate lifestyle factors that can influence hypertension, however older scales focus on medication adherence, often excluding other key domains and lacking adequate reliability and validity [3]. Managing hypertension through self-care activities such as diet, physical activity, weight management, smoking, and alcohol consumption are more effective means of controlling hypertension [4]. Although self-care assessment instruments are available [4, 5], currently there is no available instrument for use in Taiwan.

Self-care is individual actions directed toward self or the environment to regulate individual functioning, which can improve health, reduce risk, avoid related complications, and ensure one’s general well-being [4]. The Hypertension Self-Care Activity Level Effects (H-SCALE), originally developed by Warren-Findlow and Seymore [5], is used worldwide to measure self-care activities associated with lifestyle for patients with hypertension [6–10]. The H-SCALE was.

developed following the recommended guidelines of the Joint National Committee on Prevention, Detection, Evaluation, and Treatment of High Blood Pressure (JNC 7) [5]. The scale assesses six lifestyle domains that impact hypertension: medication adherence, a healthy diet, physical activity, body weight, alcohol consumption, and smoking [5, 6]. The advantage of the H-SCALE is that the assessments identify domains needing tailored interventions to improve self-care and provide a healthy lifestyle, which can reduce hypertension.

The H-SCALE has been shown to be a valid and reliable measurement of self-care activities and has been translated for application in countries spanning both Eastern and Western regions [7–10]. However, to the best of our knowledge, there is no equivalent scale that is tailored to measure self-care activities associated with a healthy lifestyle for patients with hypertension.

## Methods

### Aim

The aims of this study were to translate the Hypertension Self-Care Activity Level Effects (H-SCALE) from the English-language version [5] to a Chinese version (H-SCALE-C), adapt the translated scale for patients with hypertension, and test the reliability and validity of the scale.

### Study design and participants

The English version of the H-SCALE was translated into Chinese using a forward-back translation method, described below. Psychometric properties of the translated scale were examined thru a cross-sectional convenience sample of patients with hypertension. To include patients with a broad spectrum of hypertension experiences and management practices, the sample was recruited from a cardiovascular outpatient clinic of a medical center in northern Taiwan. Patients were eligible to participate if they met the following criteria: (1) 20–79 years of age; (2) diagnosed with primary hypertension; and (3) able to read and understand Chinese. Patients were excluded for any of the following criteria: (1) having a diagnosis of cancer, thyroid disease, or a psychiatric disorder; (2) with a history of a permanent pacemaker, heart transplant, or implantable cardioverter defibrillator; (3) currently pregnant; (4) use of antidepressants; or (5) having a history of drug or alcohol abuse.

### Original H-SCALE

The original H-SCALE, developed by Warren-Findlow et al. in 2011 and revised in 2013, includes 31 items divided into six domains: medication adherence, dietary quality, physical activity, smoking, weight management, and alcohol consumption [5, 6]. Each domain (subscale) represents a component of a patient’s self-care management, which is assessed over a period of a week or a month. The Cronbach’s alphas for the six subscales range from 0.67 to 0.86 [6]. Details of the six domains are described below.

#### Medication adherence (items 1–3)

Medication adherence assesses patient’s compliance over one week with taking a prescribed antihypertensive medication using the following three items: (1) take blood pressure medication, (2) takes it at the same time every day, and (3) takes the recommended dosage. Each item is scored from no days (0) up to everyday (7). Total scores range from 0 to 21 points. In this study, a score ≥ 17 (80%) was considered adherent.

#### Diet quality (items 4–14)

Diet quality was assessed with the revised version of the self-report scale, Dietary Approaches to Stop Hypertension (DASH). The original scale emphasized a low-sodium diet, whereas revised scale focuses on diet quality (DASH-Q) [11]. The 11-item DASH-Q scale assesses the frequency of following a healthy diet in the past 7 days, which includes avoidance of salty foods as well as the consumption of nutritionally balanced foods, such as fruits, vegetables, alternate forms of protein, and foods with potassium, fiber, and whole grains. Each item is scored from 0 to 7; total scores range from 0 to 77 points. A score greater than 52 points indicates good adherence to a healthy diet.

#### Physical activity (items 15, 16)

The physical activity subscale is comprised of two items, which assess a patient’s adherence to a recommended combination of aerobic and muscle-strengthening activity for 30 min per day over the last week. Activities include swimming, walking, weightlifting, repeated heavy lifting or pushing/pulling that are not related to housework or employment. Total scores range from 0 to 14; a score greater than 8 points indicates good adherence.

#### Smoking (items 17, 18)

Smoking exposure was assessed with two questions: “How many of the past 7 days did you smoke a cigarette (even just one puff)?” (item 17, frequency); and “How many of the past 7 days did you stay in a room or ride in an enclosed vehicle while someone was smoking?” (item 18, passive smoking exposure). Items are scored 0 to 7 days. The summed scores for the two items range from 0 to 14; lower scores indicate better adherence to self-care.

#### Weight management (items 19–28)

The subscale for weight management is comprised of 10 items that assess activities that can help manage body weight, such as reducing portion sizes or making food substitutions. Each item is a statement about the use of a weight management activity over the past 30 days, such as “I have cut out or limited some foods I like but are not good for me” (item 25). Items are scored on a 5-point Likert scale from strongly disagree (1 point) to strongly agree (5 points). Total scores range from 10 to 50; higher scores indicate better weight management practices. Participants who score ≥ 40 (agree or strongly agree on all 10 items) are considered to have good adherence to weight management.

#### Alcohol consumption (items 29–31)

The alcohol consumption subscale assesses the average intake of alcohol over the last 7 days. Item 29 asks, “On average, how many days a week do you drink alcohol?” A response = 0 indicates good adherence. However, if the response is > 0, the participant must complete items 30 and 31, which are write-in answers. Item 30 asks, “On a typical day when you drink, how many drinks do you have?” Item 31 asks, “What is the maximum number of drinks you had on any given day?” The level of non-adherence is determined by multiplying the numbers given for items 30 and 31 by the number of days reported in item 29. Moderate consumption among men is considered ≤ 2 drinks/day (a score ≤ 14); among women, ≤ 1drink/day is considered moderate (a score ≤ 7).

### Translation of the H-SCALE

Permission to translate the English-language H-SCALE was obtained from Dr. Warren-Findlow. The instrument has been translated and validated for use in other languages to measure self-care in patients with hypertension, for instance the Eastern countries of Pakistan [7] Myanmar [8], and Iran [9] and the United States [6, 9, 11].

The forward-backward method of Brislin was used to translate the H-SCALE, which ensures language equivalence (meaning) for cross-cultural translations [12]. First, a bilingual registered nurse independently translated the H-SCALE into Chinese. A second bilingual registered nurse, blinded to the original version, translated the scale from Chinese back to English. The English-language back-translated version was compared with the original version of the H-SCALE to ensure content equivalence of the translation. To further ensure that the grammar, syntax, and context of the translated scale was equivalent to the original, we conducted a group discussion with bilingual physicians and nurses from the cardiovascular department. Differences in meanings between the two languages were identified and changes were made until group consensus for equivalence of the two versions was reached.

### Content validity of the translated Chinese version scale

Five experts in the field of cardiovascular medicine assessed the content validity of the Chinese translation of the scale. Content validity determines if a translated scale is semantically and culturally equivalent to the original scale. Each expert rated the 31 translated items on a 4-point Likert scale from 1 (not at all equivalent) to 4 (completely equivalent). Items with mean scores ≤ 2 (items 3, 7, 12, 15, 16, 17, and 27) were reworded until a score ≥ 3 was obtained, indicating equivalency was achieved. For instance, item 12 included consuming the vegetable “collard greens”, which are not often eaten in Taiwan. Therefore, we replaced collard greens with “sweet potato leaves”, a vegetable similar in nutrition to collard greens and commonly eaten in Taiwan. The overall Content Validity Index (CVI) of the Chinese version of the H-SCALE (H-SCALE-C) was 0.93, which is considered acceptable.

### Item analysis

The H-SCALE-C was pilot tested with 20 outpatients with hypertension. After completing the questionnaire, patients were interviewed about clarity of the wording and ability understand the questions. Most patients reported they could not accurately answer the question about the average number of days they drank alcohol in the previous week (item 29). Because scores for non-adherence are the result of multiplying the number of drinks per day (item 30) and maximum number of drinks on a given day (item 31) by the number of days of alcohol consumption (item 29), we felt we would not be able to accurately calculate a score for alcohol consumption and the subscale was removed. Therefore, prior to factor analysis, the H-SCALE-C was comprised of 28 items and five domains.

### Data collection

Data were collected in the outpatient clinic from April 2017 to September 2017, after patients provided informed consent. Participants filled out a survey questionnaire about demographic characteristics, such as age, gender, employment. Clinical characteristics, such as duration of hypertension, smoking (yes/no), variables of body mass index (BMI), and comorbidities, were obtained from the patients’ charts, with their permission. They were then provided instructions for completing the H-SCALE-C questionnaire.

### Ethical considerations

Approval for conducting this study was obtained from Institutional Review Board of the Tri-Service General Hospital Institutional Review Board. The design and purpose of the study were explained to the patients. They were assured of confidentiality of their data, and the right to refuse to continue with the study at any time and for any reason. All participants provided informed consent and data were coded to maintain anonymity.

### Statistical analysis

The SPSS V.23 (IBM Corp, Armonk, New York, USA) was used to perform statistical analysis. Data for continuous variables were described using mean and standard deviation (SD), while categorical variables were presented with frequency and percentage. The level of significance was set to *p* < .05.

Although the English version of the H-Scale has been demonstrated to be a valid instrument for patients with hypertension, possible differences between Western and Eastern cultures prompted us to view the H-SCALE-C as a new instrument and construct validity of the scale was examined with exploratory factor analysis (EFA). The recommended minimum sample size for EFA is 5–10 participants for each item or 155 to 310 for 31 items indicating the sample of 318 participants in this study was adequate for factor analysis and determining reliability of the H-Scale-C [4, 13]. H-SCALE-C data from all 318 participants were used for EFA and subsequent analyses.

The Kaiser-Meyer-Olkin (KMO) measured the sampling adequacy and Bartlett’s test of sphericity determined suitability of the data for factor analysis. A KMO greater than 0.7 and Bartlett’s test of sphericity less than 0.05 are considered adequate to conduct factor analysis.Data extraction was implemented through principal component analysis (PCA). The factors with eigenvalues > 1.0 were retained, and factor loadings greater than 0.40 indicated that items for each factor could be retained [14].

CFA was conducted using AMOS V.17, version 22.0 (IBM Corp, Armonk, New York, USA). Fit indices for the model were considered acceptable based on the following criteria: Chi-square/degrees of freedom (*df*) < 3; goodness-of-fit index (GFI) > 0.90; adjusted goodness-of-fit index (AGFI) > 0.90; comparative fit index (CFI) > 0.90; standardized root mean square residual (SRMR) ≤ 0.05; and root mean squared error of approximation (RMSEA) ≤ 0.05 [15]. Convergent validity of the H-SCALE-C was evaluated with values for composite reliability (CR), as a measure of internal consistency, and average variance extracted (AVE), which measures the variance of a construct. Values for CR above 0.70 and AVE ≥ 0.5 are considered acceptable [15]. Discriminant validity was assessed by comparing the square root of the AVE for each factor with correlations between factors. A value for the square root of the AVE greater than correlation coefficients between factors indicates acceptable discriminant validity [15].

To ensure that individual items of the translated H-SCALE-C were culturally equivalent to the self-care activities of the original H-SCALE, items were removed if the correlation coefficients between item scores and item-to-total scores were < 0.40, which is more stringent than < 0.30 and results in stronger relationships between items and the total scale [16]. The internal consistency reliability for the H-SCALE-C was established using Cronbach’s α. A Cronbach’s α greater than 0.7 indicates satisfactory internal consistency [17].

**Fig. 1:**
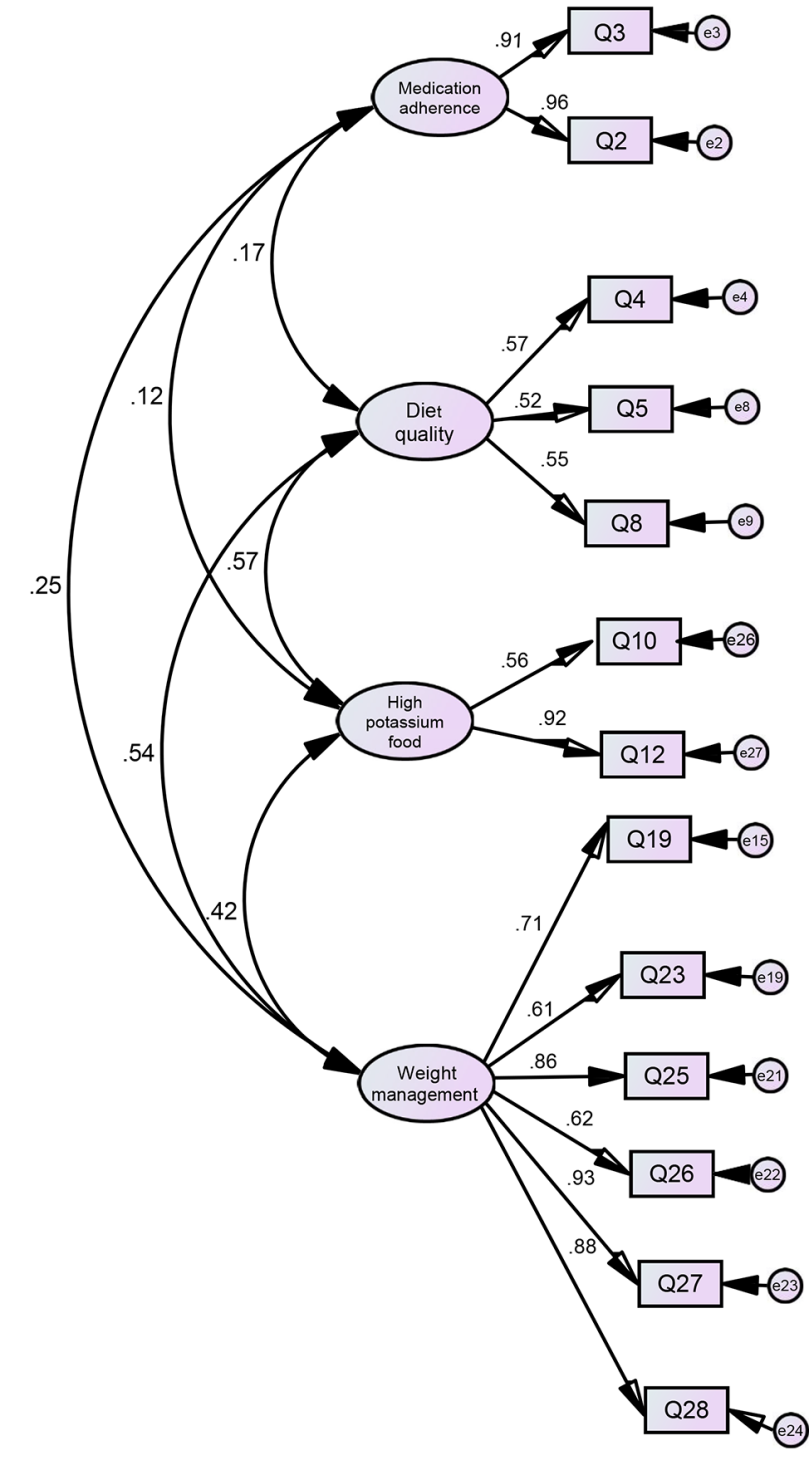
Confirmatory factor analysis of the 13-item Chinese translation of the Hypertension Self-Care Activity Level Effects (H-SCALE-C), based on the four-factor model

## Results

### Sample characteristics

A total of 318 Taiwanese patients with hypertension were recruited to evaluate the reliability and validity of the translated H-SCALE-C. The mean age of participants was 63.91± 11.80 years (range = 28 to 88 years); slightly over half were male (*n* = 170, 53.5%); most (*n* = 282, 87.0%) were married; and 37.1% had a college degree or higher. The mean duration of hypertension was 7.53 ± 6.68 years; 65.1% had hypertension for a duration of more than 5 years. The mean BMI was 26.28 ± 4.01 kg/m^2^; 41.5% (*n* = 132) had a BMI ≥ 24 kg/m^2^. Most had one or more comorbidity (67.6%); of those, 44.3% had hyperlipidemia. Other demographic and clinical characteristics of the participants are summarized in Table 1.

**Table 1:** Characteristics of participants (*N* = 318)

Characteristic	*n* (%)	Mean (SD)
Age (years)		63.91 (11.80)
Gender		
Male	170 (53.5)	
Female	148 (46.5)	
Employment status		
Employed	133 (41.8)	
Unemployed	185 (58.2)	
Educational Levels		
Less than high school	139 (43.7)	
High school	61 (19.2)	
College/university/master’s degree	118 (37.1)	
Marital status		
Married	282 (88.7)	
Single	9 (2.8)	
Divorced/ Widowed	27(8.5)	
Systolic blood pressure (mmHg)		153.87 (18.83)
Diastolic blood pressure (mmHg)		87.07 (13.23)
Duration of hypertension		7.53 (6.86)
< 5 years	111 (34.9)	
5–10 years	142 (44.7)	
> 10 years	65 (20.4)	
Smoking status		
Yes	25 (7.86)	
Body mass index (BMI) (kg/m^2^)		26.28 (4.01)
Overweight (24 kg/m^2^ ≤ BMI<27 kg/m^2^)	113 (35.5)	
Obese (BMI ≥ 27 kg/m^2^)	132 (41.5)	
Comorbidity		
0	103 (32.4)	
1	121 (38.1)	
≥ 2	94 (29.5)	
Top 2 Comorbidities		
Hyperlipidemia	141 (44.3)	
Diabetes	83 (26.1)	

*Note*: SD = standard deviation

### Factor analysis

CFA was first conducted with a maximum likelihood estimate based on the five-factor model of the H-SCALE-C. However, analysis indicated most fit indices did not meet the criteria for acceptability (Table 2).

**Table 2:** Confirmatory factor analysis of the 5-factor model and 4-factor model

		Indices						
Variables	χ^2^	df	χ^2^/df	GFI	AGFI	CFI	SRMR	RMSEA
Acceptable criterion	-	-	< 3.00	> 0.90	> 0.90	> 0.90	≤ 0.05	≤ 0.05
5-Factor model	718.56^***^	340	2.11	0.85	0.83	0.91	0.06	0.06
4-Factor model	66.07	55	1.20	0.97	0.95	0.99	0.04	0.03

Note: χ^2^=Chi-square, likelihood-ratio; df = Degrees of freedom; GFI = Goodness-of-fit index; AGFI = Adjusted goodness-of-fit index; CFI = Comparative fit index; RMSEA = root mean squares error of approximation; SRMR = Standardized root mean square residual

*** *p* < .001

Therefore, EFA was conducted to determine construct validity of the scale. The KMO was 0.85 and Bartlett’s test of sphericity achieved significance (*p* < .001), indicating factor analysis was appropriate. Fifteen items were deleted following EFA due to factor loadings below 0.40, which included one item for medication adherence (recommended medication), four for weight management, both items for physical activity, both items for smoking, and six items for diet quality, which included consumption of eggs and high-sodium foods. PCA with Varimax rotation extracted four factors for the 13 items, which were comprised of three of the five original domains plus an additional domain, which we labeled “high potassium food”. All items met the assumption of normality [18] and multivariate normality was established with Mardia’s coefficient [19]. There was no cross-loading for any of the factors (Table 3). The new factor consisted of two items previously included in the diet quality subscale. The four extracted factors explained 63.93% of the total variance: 34.42% from weight management (six items); 12.32% from medication adherence (two items); 9.36% from diet quality (three items); and 7.84% from high potassium food (two items). Convergent validity was good, as demonstrated by a CR greater than 0.60 and an AVE ≥ 0.40. The overall Cronbach’s alpha value was 0.76. Cronbach’s alpha coefficient for each domain was between 0.65 and 0.94.

**Table 3:** Exploratory factor analysis and normality for the 13 items of the Chinese version of the Hypertension Self-Care Activity Level Effects Scale (*N* = 318)

Original					Factor loadings					
Item #	Factor and new item #	Mean (SD)	Skewness	Kurtosis	1	2	3	4	CR	AVE
**Weight management**									0.89	0.57
27.	**1**. I substitute healthier foods for things I used to eat.	3.07 (1.09)	-0.49	-0.54	0.87					
28.	**2**. I have modified my recipes when I cook.	3.09 (1.11)	-0.54	-0.55	0.87					
26.	**3**. I eat at restaurants or fast-food places less often.	2.87 (1.27)	-0.31	-1.20	0.78					
25.	**4**. I have cut out or limited some foods I like but that are not good for me.	2.92 (1.23)	-0.31	-0.68	0.71					
19.	**5**. I am careful about what I eat.	2.66 (1.29)	-0.10	-1.00	0.66					
23.	**6**. I eat smaller portions, or I eat fewer portions.	2.79 (1.22)	0.02	-1.18	0.63				0.95	0.91
**Medication adherence**										
3.	**7**. I take the recommended dosage of blood pressure pills.	6.28 (1.80)	-2.76	5.57		0.97				
2.	**8**. I take my blood pressure pills at the same time every day.	6.11 (1.92)	-2.32	4.33		0.94				
**Diet quality**									0.72	0.47
5.	**9**. Do you eat beans, peas or lentils?	3.08 (2.45)	0.52	-1.05			0.81			
4.	**10**. Do you eat nuts or peanut butter?	1.61 (2.37)	1.38	0.53			0.69			
8.	**11**. Do you eat five or more servings of fruits or vegetables?	2.08 (2.93)	0.89	-1.00			0.53			
**High potassium food**									0.79	0.65
10.	**12**. Do you eat more than one serving of a vegetable?	6.32 (1.57)	-2.37	4.08				0.84		
12.	**13**. Do you eat broccoli, sweet potato leaves, potatoes, squash, or sweet potatoes?	4.54 (2.24)	-0.25	-1.23				0.77		
Multivariate normality			61.41							
Explained variances (%)					34.42	12.32	9.36	7.84		
Cronbach’s alpha					0.89	0.94	0.65	0.66		

*Note*: SD = standard deviation; CR = composite reliability; AVE = average variance extracted; multivariate normality established with Mardia’s coefficient

Fit indices from CFA of the 13-item 4-factor H-SCALE-C demonstrated an adequate model fit (Table 2; Figure 1). Table 4 shows the model had good discriminant validity, as demonstrated by the value for the square root of the AVE for the four factors being greater than all correlations among the factors [15].

**Table 4:** Discriminant validity of the 4-factor Chinese translation of the Hypertension Self-Care Activity Level Effects (H-SCALE-C) (*N* = 318)

Factor	1	2	3	4
1. Weight management	**(0.75)**			
2. Medication adherence	0.26	**(0.95)**		
3. Diet quality	0.39	0.12	**(0.69)**	
4. High potassium food	0.39	0.12	0.36	**(0.81)**

*Note*: Values in bold on the diagonal in parentheses represent the square root of the average variance extracted (AVE) and the values outside represent the correlations between factors

## Discussion

The new H-SCALE-C was translated from the H-SCALE developed by Warren-Findlow et al. [5, 6], which contains 31 items and six domains. Although the content of the translated 31 items had equivalence with the original items and the CVI was acceptable, item analysis resulted in the removal of the domain of alcohol. EFA with PCA of the 13 remaining items extracted four factors, which eliminated the domains of physical activity and smoking. The final H-SCALE-C is a 13-item instrument with three of the original domains (weight management, medication adherence, diet quality) and a new domain labeled low sodium food.

The primary strength of the original English version of the H-SCALE lies in its coverage of lifestyle domains for patients with hypertension recommended by JNC-7 [5, 6], which can enable healthcare providers to swiftly assess the activity levels for reducing hypertension and provide them with timely suggestions relevant to their self-care needs. The participants who completed the H-SCALE-C for factor analysis represented a wide range of characteristics for patients with hypertension. These patients included young and older adults, diagnosed with hypertension recently, living with hypertension for > 10 years, and with and without comorbidities. Therefore, responses to the questionnaire represented a broad range of compliance with self-care management activities to control hypertension. Although only three of the original domains reflected activities our participants employed to manage hypertension, we believe the data provided by this comprehensive group of Taiwanese patients will allow clinicians to reference HSCALE-C assessments when providing patients with individualized self-care strategies that will help them achieve modifications for a healthy lifestyle.

One disadvantage of the H-SCALE-C is the absence of the assessment of alcohol consumption, smoking, and physical activity, all of which are considered important by the JCN-7 guidelines for blood pressure control [20]. The absence of the domain of smoking as a factor in the HSCALEC may be linked to cultural influences, which might also explain the difficulty the pilot group had when assessing the amount of alcohol consumed. Participants may not have recognized refraining from smoking as an important part of self-care for reducing hypertension. Both alcohol and smoking are important components of societal interactions in Chinese cultures [21, 22], where signs of friendliness are frequently demonstrated by proposing a toast with an alcoholic drink or offering someone a cigarette [23]. The exclusion of the domain for physical activity from the H-SCALE-C might also be the result of cultural influences. Rio and Saligan (2023) reported that both cultural and contextual factors can influence an individual’s attitude towards physical activity. For instance, physical activity may not be considered a health benefit if it is viewed as a leisure activity [24]. A scale for self-care for Chinese patients with hypertension in Hong Kong developed by Ma et al. (2020) addressed the negative influence of cultural barriers on drinking and smoking by placing items related to these behaviors under the category of ‘habit modification’ [23]. Hence, modification of the H-SCALE-C by including items related to drinking, smoking and physical activity under the domain of ‘habit modifications’ for self-care will be explored in future studies.

The four domains identified (high potassium foods, weight management, medication adherence, and diet quality) are important self-care activities related to lifestyle in patients with hypertension. Nuclear families remain important in the culture of Taiwan and have a significant influence on the habits of patients in Eastern societies, which differs from Western cultures [21]. One important component of Asian families is the emphasis on group harmony, which makes dietary options, including cooking methods, not only personal choices but also require considering the expectations of family [23]. Therefore, when designing strategies to help patients with hypertension modify disease management strategies involving food consumption and daily activities, nurses should also include feedback from family members as to what might be most helpful for improving self-care behaviors. A study on self-care for Korean Americans with diabetes reported that adherence to self-management was positively influenced by support from family members [25]. The influence of families on patients with diabetes self-care behaviors was also important for individuals who had immigrated from the Middle East to the US [26].

### Limitations

This study had some limitations. First, participants were recruited by convenience sampling from one cardiovascular outpatient clinic of a medical center in northern Taiwan, which restricts the external validity of this study. Therefore, further studies should enroll patients from multiple regions of Taiwan to determine the generalizability, confirm the validity, and strengthen the reliability of the H-SCALE-C. Second, limited time and resources prevented us from evaluating test–retest reliability of the H-SCALE-C, therefore we do not know if the findings are stable over time. We plan to examine this reliability in future studies. Third, the absence of an evaluation of the domains of alcohol consumption, smoking, and physical activity prevent a complete assessment of behaviors known to increase risk of cardiovascular complications for patients with hypertension. Finally, there was no comparison of scores on the H-SCALE-C with another self-care instrument for patients with hypertension, which resulted in a lack of comparative analysis.

## Conclusion

The Chinese version of the H-SCALE was demonstrated to be a reliable and valid measure of self-care abilities, which could be applied as a means to facilitate lifestyle modifications to manage hypertension for patients in Taiwan. The higher prevalence of hypertension in Taiwan makes it critically important for access to a suitable instrument to assess self-care activities for patients with hypertension. The H-SCALE-C should be considered for use in clinical practice as an effective means of quickly assessing self-care abilities for patients with hypertension in Taiwan. Scale scores could be used by clinical nurses to provide tailored interventions for lifestyle changes that improve blood pressure control. Healthcare providers could use HSCALEC assessments as a record of quantitative changes in self-care activities, allowing providers to accumulate evidence about the impact of lifestyle changes on blood pressure management for patients with hypertension in Taiwan. Adding an additional domain for ‘habit modification’ that includes alcohol consumption, smoking, and physical activity could further strengthen the applicability of the H-SCALE-C.

## Data Availability

There are no unpublished data from this study. To access the dataset can contact the.

## Abbreviations

H-SCALE: Hypertension Self-Care Activity Level Effects
CVI: Content validity index
H-SCALE-C: Chinese version of the H-SCALE
CFA: Confirmatory factor analysis
EFA: Exploratory factor analysis
WHO: World Health Organization
JNC 7: Joint National Committee on Prevention, Detection, Evaluation, and Treatment of High Blood Pressure
DASH-Q: Dietary Approaches to Stop Hypertension-Quality diet
DASH diet: Dietary Approaches to Stop Hypertension diet
BMI: Body mass index
SD: Standard deviation
KMO: Kaiser-Meyer-Olkin
PCA: Principal Component Analysis
GFI: Goodness-of-fit index
AGFI: Adjusted goodness-of-fit index
GFI: Comparative fit index
RMSEA: Root mean squared error of approximation
CR: Composite reliability
AVE: Average variance extracted
